# Multimodality Management of Ruptured Large Hepatocellular Carcinoma and Its Recurrence: Rupture at Presentation Should Not Rupture Hope of Long-Term Survival

**DOI:** 10.1055/s-0040-1710530

**Published:** 2020-06-16

**Authors:** Gunjan S. Desai, Prasad M. Pande, Rajvilas A. Narkhede, Prasad K. Wagle

**Affiliations:** 1 Department of Surgical Gastroenterology, Lilavati Hospital and Research Centre, Mumbai, Maharashtra, India; 2 Department of Surgical Gastroenterology, Dr. Balabhai Nanavati Superspeciality Hospital, Mumbai, Maharashtra, India

**Keywords:** sorafenib, hepatocellular carcinoma, trisectionectomy, portal vein embolization

## Abstract

A 59-year-old gentleman with a history of aortic valve replacement presented with spontaneously ruptured hepatocellular carcinoma in right lobe of a hepatitis C virus (HCV)-related chronic liver disease with hemoperitoneum. This acute emergency was managed by transarterial embolization. Right trisectionectomy with preservation of segment IVB after augmentation of future liver remnant by transarterial chemoembolization followed by portal vein embolization was subsequently performed. Sustained virological response to HCV was attained after surgery using sofosbuvir-based regimen. He had a delayed operative bed recurrence 1.5 years later with pulmonary metastatic disease which was managed by operative bed metastasectomy with mesh reconstruction of diaphragm and sorafenib. He is on sorafenib since past 3 years and doing well at 4.5-years follow-up since the first presentation, with significant regression of pulmonary disease and no other disease elsewhere, which highlights that where there is hope, there is a way.

Spontaneous rupture of a 12-cm hepatocellular carcinoma (HCC) in a hepatitis C virus (HCV)-infected cirrhotic liver with hemoperitoneum is rare, grave, and a tricky situation to manage due to lack of established standard guidelines. 1 2 3 We present here a case that survived this situation, had a delayed local recurrence with metastatic disease, managed by second surgery and sorafenib, doing well at 4.5 years follow-up since the first presentation, which highlights that where there is hope, there is a way.

## Case Report

A 59-year-old gentleman presented in the emergency department with a sudden onset of abdominal pain, breathlessness, and dizziness, with a history of aortic valve replacement (1980) and on tablet warfarin. He had tachycardia, pallor, generalized abdominal distension, and tenderness of abdomen.

**Fig. 1:**
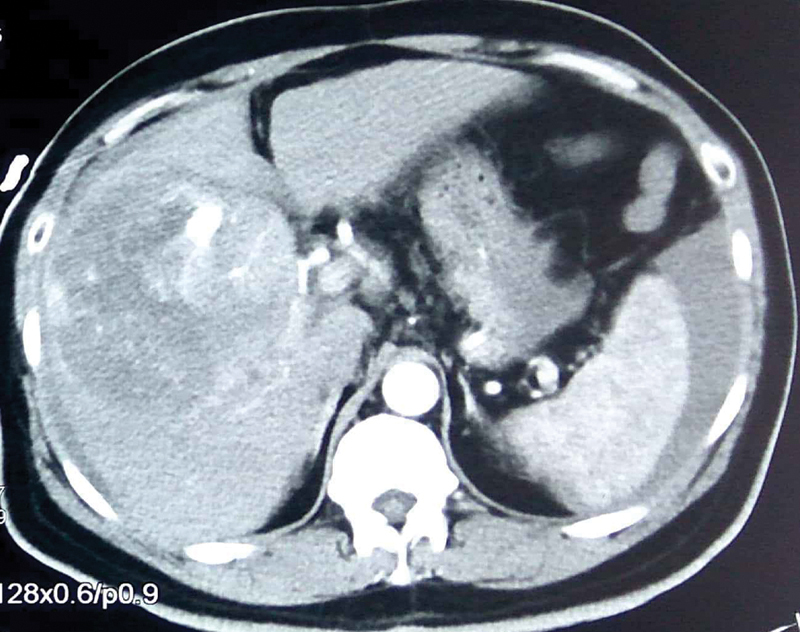
Contrast enhanced computed tomography axial image shows hemoperitoneum, arterially enhancing lesion in liver (hepatocellular carcinoma), and an intratumoral aneurysm with contrast blush.

Hemoglobin was measured to be 6 g/dL. Contrast enhanced computed tomography scan of abdomen revealed a 12 × 9 × 9-cm heterogeneous lesion with arterial enhancement and portal phase washout suggestive of HCC, intratumoral contrast pooling, and blush suggestive of intratumoral pseudoaneurysm with rupture and hemoperitoneum ( Fig. 1 ), without any extrahepatic disease.

**Fig. 2:**
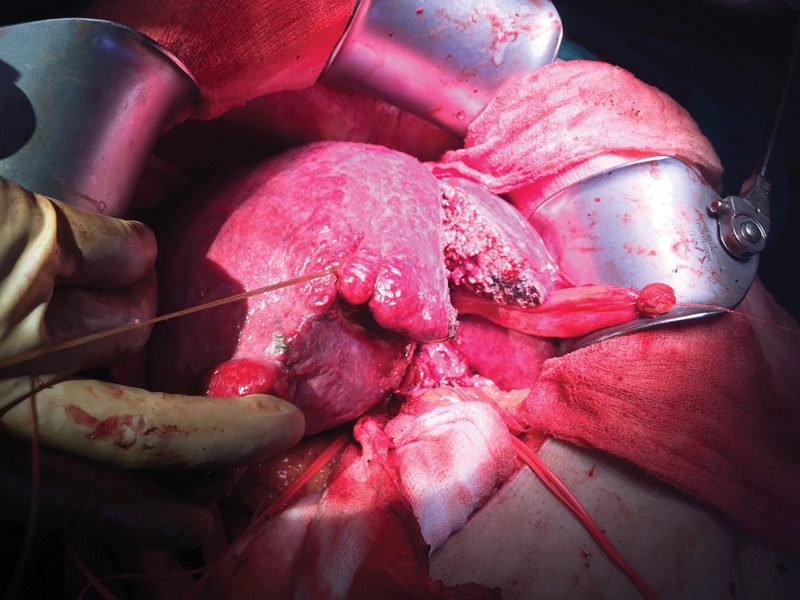
Intraoperative image showing the transaction of liver preserving segment IV B during the performance of modified right trisectionectomy.

**Fig. 3:**
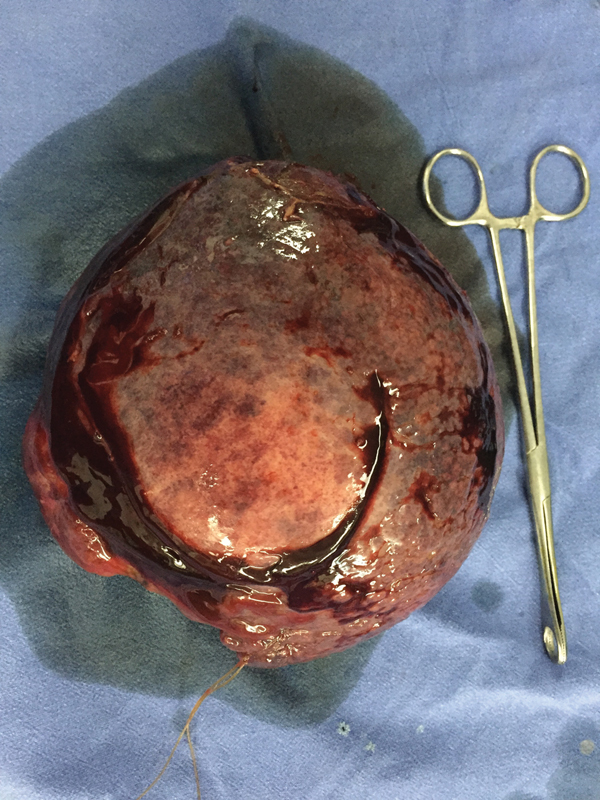
Gross tumor appearance after resection.

Angioembolization was performed. Work-up after stabilization revealed HCV infection without derangement of liver function tests. There were no esophageal varices, no splenomegaly, and normal platelet counts. Portal vein Doppler was normal, and no collaterals were present. serum alpha-fetoprotein (AFP) was 8.7 ng/mL. Metastatic disease was ruled out by a high-resolution chest CT. A right trisectionectomy was warranted. CT liver volumetry revealed future remnant liver volume (FLR) of 23%. Hence, a transarterial chemoembolization (TACE) followed by portal vein embolization (PVE) was performed. FLR of 41% was achieved at 6 weeks. He underwent right trisectionectomy ( Figs. 2 , 3 ), recovered uneventfully, and was discharged on postoperative day 8. Histopathology revealed a moderately differentiated HCC with negative margins and background liver fibrosis METAVIR score F3. Sofosbuvir-based regimen was administered for 12 weeks and sustained virological response (SVR) achieved.

**Fig. 4:**
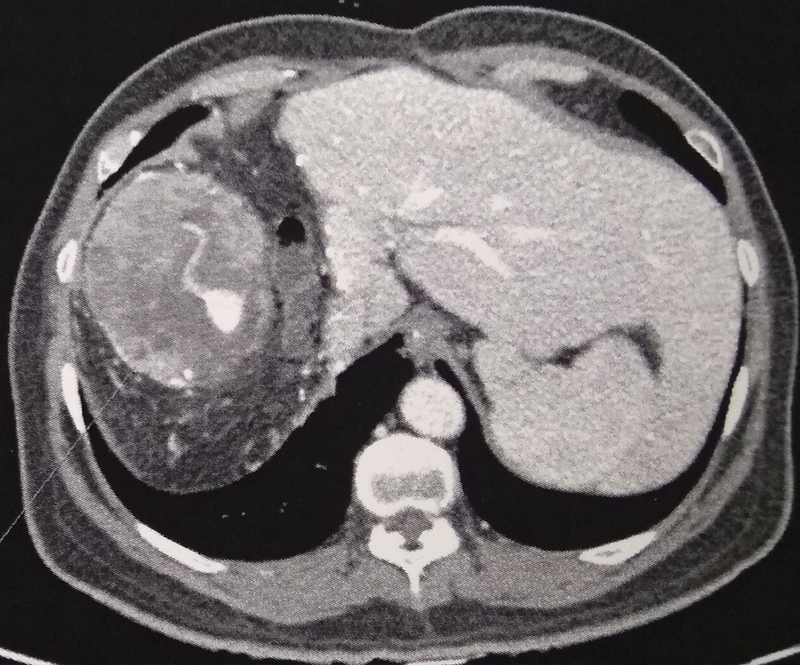
Contrast enhanced computed tomography axial image shows an intra-abdominal 10 × 9 × 9-cm lesion adherent to diaphragm and the operative bed along the cut surface of liver.

**Fig. 5:**
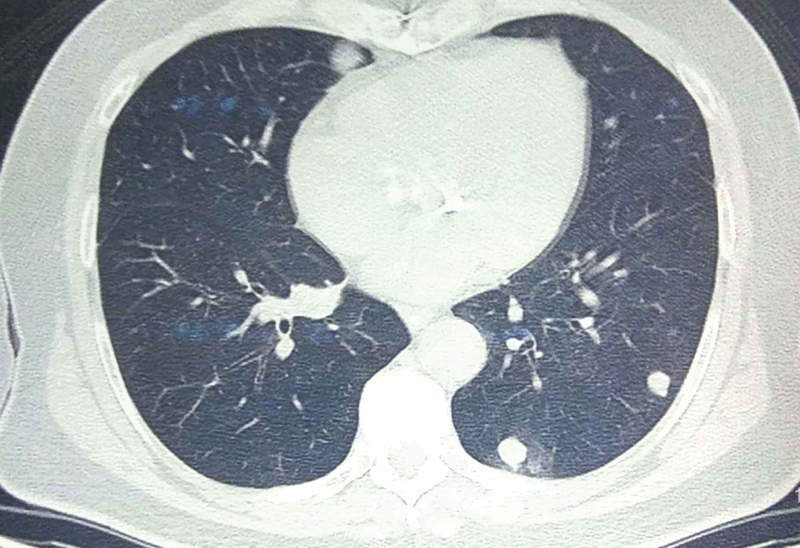
Positron emission tomography–computed tomography scan thorax shows lesions in left lung field.

He was disease-free, on regular follow-up for 18 months, with normal imaging and AFP levels, when he developed bilateral pulmonary metastasis with a total of five lesions, the largest size being 1.4 × 1.3 cm, with a metachronous intra-abdominal 10 × 9 × 9 cm lesion adherent to diaphragm and the operative bed with no other extrahepatic abdominal disease ( Figs. 4 , 5 ). In view of the performance status 1, limited operative bed recurrence that did not need any further liver resection, a resection of the recurrence with mesh reconstruction of diaphragm was performed. He recovered uneventfully, and sorafenib 800 mg/d was started.

He developed mucositis and diarrhea, requiring a dose reduction of sorafenib. He tolerated alternate day dosing of 800 mg/d and 600 mg/d and is on this dosing schedule for 3 years. He has not developed any further adverse reaction to sorafenib, including hand-foot syndrome.

**Fig. 6:**
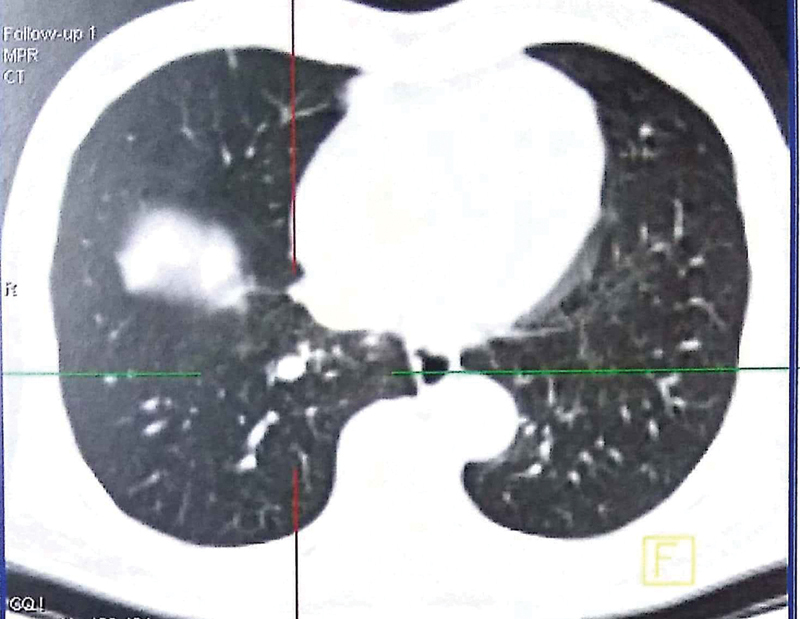
Positron emission tomography–computed tomography scan thorax shows resolution of lesions in left lung field. A single lesion is seen in the right lobe which has also reduced in size.

On follow-up positron emission tomography scan done recently, 3 years after starting him on sorafenib, and 4.5 years after his first presentation, he has near complete metabolic and morphological response wherein, the lung lesions have become non-FDG avid and have reduced in number, with no new lesions anywhere ( Fig. 6 ). His performance status and liver function are well maintained and disease controlled on sorafenib till present date, 4.5 years from his first presentation.

## Discussion

Spontaneous rupture of a large HCC is not very common. Rupture is documented in 3 to 15% cases of HCC and mortality rate is 25 to 75% across published cases. 2 3 4 Angioembolization is the preferred treatment, as performed in our patient with successful outcome and upfront surgery should only be performed in cases of failed embolization or its nonavailability. 4 5 6

Literature is sparse in this specific scenario and the long-term management of a patient with ruptured large HCC is difficult due to this reason. The systematic review by Moris et al is the largest literature review on this topic. 7 Out of the 4,941 patients included, 1,118 (22.6%) were managed conservatively, 1,601 (32.4%) underwent transarterial embolization (TAE)/TACE, 535 (10.8%) underwent emergency hepatectomy, and 897 (18.2%) underwent staged hepatectomy. Only 34% patients who managed conservatively survived beyond a month, whereas 70% patients who underwent TAE/TACE had survival beyond a month. The only treatment that offered a higher than 90% short-term survival was surgery whether emergent or elective. Patients managed by a management plan that did not include surgery had worse long-term outcomes. In this regard, staged elective surgery had the best outcomes in this systematic review.

As shown in Table 1 , other case–control and cohort studies also show that in cases with tumor rupture, staged hepatectomy gives a better outcome in terms of overall survival (OS), though the recurrence-free survival (RFS) is adversely affected by tumor rupture. 8 9 10 11 12 13 14 15 As we can see, TAE has been used by many studies for stabilization followed by elective surgery and this plan gives a better 5-year OS and RFS. Thus, the studies do point toward a good outcome in operable patients of ruptured HCC by this strategy. However, there are a few limitations which affect the interpretation of this data and patient management.

**Table 1:** Review of studies on ruptured hepatocellular carcinoma and interpretation of outcomes across these studies

Number	Number of patients (rHCC)	Number of cirrhotic patients	Tumor size >5 cm	TAE/TACE (%)	Number of patients operated	Surgery—emergency (A)	Surgery—elective (B)	Mortality	5-y RFS	5-y OS	Interpretation
1 7	4,941 (67 studies)	2,414	NA	32.4	1,432	10.8%	18.2%	NA	8.6% (A) vs. 13.1% (B)	25.7% (A) vs. 26.5% (B)	• Tumor size >5 cm, advanced cirrhosis, age, portal hypertension risk factors for tumor rupture. • Advanced cirrhosis, shock at presentation, high AFP, portal vein invasion portend poor prognosis in rHCC. • TAE followed by staged hepatectomy offers best RFS/OS.
2 ^8^	106	89	75	NA	106	8 (7.5%)	98 (92.4%)	1%	31.3%	14.7%	• Tumor rupture influences OS but not RFS after hepatectomy.
3 9	14	11	NA	57	14	7%	93%	NA	NA	16.8%	• Curative resection most important prognostic factor for rHCC.
4 10	172	164	NA	68	93	2.9%	51.1%	NA	NA	45%	• Staged hepatectomy offers better survival than TACE alone.
5 11	138	58	NA	62	24	24 (18%)	0	17%	NA	NA	• High mortality after emergency surgery.
6 12	58	18	27	71	58	5 (8.6%)	53 (91.3%)	60% (A) vs. 7.5% (B)	35%	48%	• No difference in RFS/OS due to tumor rupture. • Staged hepatectomy—better outcomes.
7 13	131	85	NA	NA	131	55.7%	44.2%	11% (A) vs. 0% (B)	10.9% (A) vs. 27.6% (B)	23.3% (A) vs. 41.4% (B)	• Better RFS/OS and lower mortality after elective staged hepatectomy than emergency hepatectomy in rHCC.
8 14	64	64	16	61	16	6.2%	18.75%	NA	NA	24%	• Tumor rupture shortens RFS. • Significant extrahepatic recurrences.
9 15	119	50	NA	44	21	2.5%	15.1%	NA	35.5%	55.2%	• TACE alone has poorer survival than staged hepatectomy.

Abbreviations: AFP, alpha-fetoprotein; OS, overall survival; RFS, recurrence-free survival; rHCC, ruptured hepatocellular carcinoma; TACE, transarterial chemoembolization; TAE, transarterial embolization.

Apart from the systematic review by Moris et al, all studies are retrospective in nature. Most studies have not mentioned the tumor size, and among those that have mentioned, only three studies have ruptured large HCC (>5 cm). These studies report a shorter RFS, but, do not comment on how the recurrences/metastases have been managed and the role of systemic therapy in this setting. Our case highlights the importance of discussion of these factors after successful management of ruptured HCC. We recommend resection for operable recurrence in otherwise fit patient with healthy liver and systemic therapy after the management of scar metastasis, especially when extrahepatic metastases are also present.

The other issue in our case was the size of HCC leading to inadequate FLR. In such cases, the options are to downsize the tumor by TACE/transarterial radioembolization (TARE) or facilitate the growth of FLR by PVE/associating liver partition and portal vein ligation for staged hepatectomy (ALPPS), or a combination of these procedures. 16 17 18 TACE followed by PVE is being preferred in recent studies especially for large HCC in fibrosis, cirrhosis, steatotic, or steatohepatitic livers. 18 19 A study has published compulsory TACE followed by PVE before major hepatectomy in chronic liver disease with HCC to select livers with good regenerative capacity. Also, the median duration of TACE-PVE is 30 days (range 9–120 days) and the median duration of PVE-surgery is 28 days (range 21–45 days) thus, showing that the sequential approach does not delay resection significantly. 18 19 20 We prefer the sequential TACE followed by PVE approach, as can be seen in our case.

The accepted SVR of direct acting antivirals (DAAs) was found to be 95% and they became the preferred regimen in HCV treatment surpassing the interferon-based regimens. 21 However, a lot of studies since 2016 have raised a concern on a high incidence of early occurrence and recurrence of HCC after treatment with DAAs. 22 23 Recent review have concluded that the question whether DAA promotes tumor growth is still unanswered and the association is not proven to be causative as of now. 24 25 Our patient was also treated with DAA-based regimen and he had a delayed recurrence (at a follow-up duration of 18 months) which does not match the risk factor time group of these studies.

Sorafenib is the drug of choice in inoperable and metastatic HCC patients with good performance status and acceptable liver function. 26 27 However, sorafenib as an adjuvant treatment in the prevention of recurrence of HCC (STORM) trial was not found to be beneficial. Many other trials in this line found similar results and hence, sorafenib is not preferred in adjuvant setting. 28

The major issues with sorafenib are its long-term tolerability, cost, and an undefined end point of treatment when it is used in metastatic and advanced HCC. The median duration of starting sorafenib since the diagnosis of HCC is 15.9 months and the usual indications include disease progression or lack of response on TACE/TARE, or appearance of metastatic disease or inoperable HCC in a patient with good liver function and performance status. 29 30 Dose modifications are frequent and lower doses do not affect effectiveness according to the global investigation of therapeutic decisions in HCC and of its treatment with sorafenib (GIDEON) registry. 31 In our case, we started sorafenib at duration of 18 months from diagnosis, modified dose due to presence of diarrhea and mucositis and he is on sorafenib for 3 years now.

There are no established guidelines on the maximum duration of sorafenib therapy and the end points to stop sorafenib therapy. The most common reason to stop it is disease progression and other reasons include intolerability, decreased liver function, or noncompliance. Our patient has tolerated sorafenib so far and is on this treatment.

## Conclusion

Ruptured large HCC by itself is not a contraindication for surgery, but better outcomes are seen with transarterial embolization for hemostasis followed by planned staged hepatectomy in patients with good performance status and preserved liver function. TACE followed by PVE is the preferred method for FLR augmentation to facilitate curative resection in cases with large HCC and inadequate FLR. Upfront resectable local recurrence should be operated to negative margins in appropriately selected cases. Sorafenib is the drug of choice in case of disease progression or metastasis after curative resection.
